# A biodegradable antimicrobial oligomer-containing hydrogel for drug-resistant bacteria-infected skin wound treatment

**DOI:** 10.1016/j.pscia.2025.100091

**Published:** 2025-08-22

**Authors:** Min Wang, Xinyun Zeng, Xiuping Wang, Zhiyuan Zhang, Siwei Guo, Yang Deng, Xin Li, Lin Yao, Jiaqi Li, Wing-Leung Wong, Yugang Bai, Xinxin Feng

**Affiliations:** a Institute of Chemical Biology and Nanomedicine, State Key Laboratory of Chemo and Biosensing, Hunan Provincial Key Laboratory of Biomacromolecular Chemical Biology, and Department of Chemistry, Hunan University, Changsha, 410082, China; b Department of Pharmacy, The Third Hospital of Changsha, Changsha, 410082, China; c Department of Medicinal Chemistry and Pharmaceutical Analysis, School of Pharmacy, Air Force Medical University, Xi’ an, 710032, China; d Department of Applied Biology and Chemical Technology, The Hong Kong Polytechnic University, Hung Hom, Kowloon, 999077, the Hong Kong Special Administrative Region of China

**Keywords:** Antimicrobial hydrogel, Wound infection, Membrane disruption, DNA targeting, Reactive oxygen species

## Abstract

Antibiotic resistance poses a serious global threat, contributing to severe clinical outcomes such as skin and soft tissue infections. Effective treatment of these infections requires both potent antimicrobial activity against resistant pathogens and wound dressings that can conform closely to the wound site. Degradable antimicrobial polymers offer a promising solution to this challenge. Unlike traditional antibiotic-loaded dressings, which often fail against multidrug-resistant (MDR) bacteria, antimicrobial polymers can effectively overcome resistance barriers. Moreover, these polymers can be easily incorporated into wound dressing materials—hydrogels being a particularly advantageous platform due to their biocompatibility and wound-conforming properties. In this study, we developed a modular strategy to integrate a biodegradable cationic antimicrobial oligomer, oligoamidine (**OA1**), into a thermo-responsive hydrogel. **OA1** exerts a triple antibacterial mechanism involving membrane disruption, DNA binding, and ROS generation. The resulting hydrogel system can be conveniently formulated by simple mixing and undergoes a solution-gel transition at body temperature, enabling easy application to infected skin wounds. Importantly, the hydrogel matrix does not impair the bactericidal efficacy of **OA1**, preserving its full antimicrobial potential. This synergistic system offers an effective and user-friendly approach for treating wounds infected with MDR pathogens.

## Highlights

• **OA1** shows triple-targeting mechanisms to defeat MDR bacteria.
• **OA1**-PF127 hydrogel enhances biocompatibility without reducing its antibacterial activity.
• **OA1**-PF127 hydrogel demonstrates high effectiveness against MDR-infected wound in a pig skin *ex vivo* infection model.

## 1 Introduction

The skin serves as a vital physical barrier against pathogens, allergens, and pollutants [1,5]. Yet, wounds frequently require disinfection to prevent bacterial infections^[^[2], [3], [4]^]^. Wound healing is influenced by multiple factors and can be significantly impaired, posing a major clinical challenge [5,6]. Chronic wounds in diabetic or immunocompromised patients, especially when infected, are particularly difficult to treat [7,8]. Bacterial colonization delays healing by sustaining inflammation and tissue damage [3,4]. The issue worsens with multi-drug resistant (MDR) bacteria, as conventional antibiotics often fail [9], particularly the nefarious MDR “ESKAPE” pathogens including *Enterococcus faecium* (*E. faecium*), *Staphylococcus aureus* (*S. aureus*), *Klebsiella pneumoniae* (*K. pneumoniae*), *Acinetobacter baumannii* (*A. baumannii*), *Pseudomonas aeruginosa* (*P. aeruginosa*), and *Enterobacter species* [[10], [11], [12]].

Cationic antimicrobial polymers and oligomers have attracted considerable interest for combating antibiotic-resistant bacteria [13,14]. Their positive charge enables interaction with and disruption of negatively charged bacterial membranes, causing cell death [15]. This membrane-targeting mechanism confers broad-spectrum activity while overcoming drug resistance^[^[16], [17], [18]^]^. However, their therapeutic potential is limited by off-target effects on eukaryotic membranes, compromising biocompatibility [19,20]. Many approaches have been taken to tackle this issue, including adjusting molecular weight, alkyl chain length, and the hydrophilic-to-hydrophobic ratio to optimize performance and biocompatibility^[^[21], [22], [23], [24], [25], [26]^]^. Recently, improving biodegradability has emerged as a key strategy to enhance biocompatibility.

In 2022, Tang et al. developed glutathione-degradable polysulfides via lipoic acid polymerization [27]. These polymers demonstrated potent antibacterial activity while reducing accumulation/toxicity risks through glutathione-triggered degradation. Our group recently designed a triple-targeting degradable oligoamidine with enhanced biocompatibility via glutathione-responsive breakdown [28]. These examples confirm that biodegradability optimization can effectively reduce polymer toxicity.

These degradable polymers show promise for treating wound infections, especially those caused by resistant bacteria. While antibiotics in wound dressings^[^[29], [30], [31]^]^, they are often fail against resistant strains, antimicrobial polymers can overcome this limitation. They can be easily incorporated into dressings like hydrogels, which provide drug-loading versatility and may enhance therapeutic efficacy^[^[31], [32], [33]^]^. Yang et al. developed a biodegradable polycarbonate hydrogel with tunable charge/molecular weight, demonstrating broad-spectrum activity [34]. However, its direct crosslinking formation limited the modularity typical of antibiotic-loaded hydrogels [35,36].

**Fig. 1:**
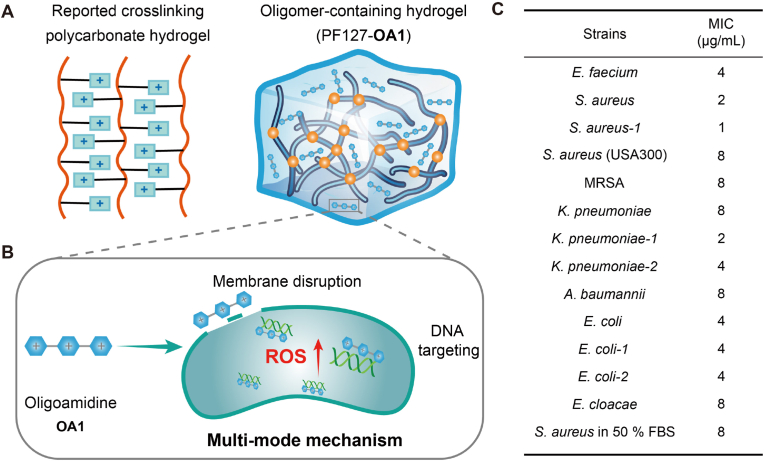
A: A schematic presentation of conventional immobilized crosslinked hydrogels and our designed thermosensitive PF127-**OA1** hydrogel with tunable drug-loading capacity; B: Scheme for an antibacterial oligomer-based hydrogel with a multi-mode mechanism including membrane disruption, DNA targeting and ROS damage; C: Antimicrobial activity of **OA1** against different bacteria.

In the present study, we developed a different approach to integrate antimicrobial polymer and hydrogel system in a modulable manner. We demonstrated that a biodegradable cationic antimicrobial oligomer, oligoamidine 1 (**OA1**), which exerts triple antibacterial effects: membrane disruption, DNA targeting, and reactive oxygen species (ROS) generation can be effectively incorporated into a thermo-responsive hydrogel for the treatment of wound infections (Fig. 1A and B). This easily prepared hydrogel forms via simple mixing and undergoes sol-gel transition at body temperature, enabling convenient application. Crucially, the hydrogel maintains **OA1**'s triple-action antibacterial efficacy against MDR pathogens.

## 2 Materials and methods

All chemicals and solvents were of analytical grade and used as received unless otherwise noted. Reagents were sourced from commercial suppliers (Shanghai Adamas Reagent, Ltd.; Shanghai Macklin Biochemical Co., Ltd.), while organic solvents were rigorously dried over activated 4 ​Å molecular sieves under inert atmosphere prior to use. Cell culture media, including Dulbecco's Modified Eagle Medium (DMEM), and fetal bovine serum (FBS) were procured from HYCLONE® (USA) and TIANHANG Biotech (China). The sheared DNA (herring sperm-derived) used in this study was obtained from Sigma-Aldrich (Shanghai) Trading Co., Ltd. Ultrapure water was generated using a Milli-Q Integral water purification system. Detailed instrument specifications and additional experimental procedures are provided in the Supporting Information.

All experiments involving pathogenic bacteria must be conducted in a Biosafety Level 2 (BSL-2) or higher containment facility.

### 2.1 Minimum inhibitory concentration (MIC) measurement

A bacterial overnight culture grown in CAMHB was diluted 1000-fold into fresh medium and incubated at 37 ​°C until reaching mid-logarithmic phase, corresponding to an optical density at 600 ​nm (OD_600_) of approximately 0.3. The culture was then further diluted in fresh CAMHB to a final concentration of 5 ​× ​10^5^ ​colony-forming units (CFU)/mL to prepare the working suspension. Aliquots of 100 ​μL were dispensed into each well of a sterile 96-well microtiter plate. The compound **OA1** was added at defined initial concentrations, followed by serial two-fold dilutions across eight consecutive wells using the same bacterial suspension. Plates were incubated at 37 ​°C for 16–24 ​h with continuous shaking at 220 ​rpm. Bacterial growth was assessed by measuring OD_600_, and the MIC was determined accordingly. All assays were conducted in triplicate or more.

### 2.2 Preparation of PF127-**OA1**

A certain mass of **OA1** was weighted in a centrifuge tube, and PF127 solution of a specific concentration and volume (pre-cooled at 4 ​°C) was added. The mixture was sonicated until **OA1** was fully dissolved, and the solution was stored in a 4 ​°C refrigerator for later use.

### 2.3 PF127-**OA1** antibacterial experiment

At 4 ​°C, 100 ​μL of PF127-**OA1** (PF127 ​= ​20% w/w) containing **OA1** of different concentrations was added to the wells of a 96-well plate. The plate was placed in a 37 ​°C incubator for 5 ​min to allow gelling. Bacterial solution of interest (100 ​μL, 5 ​× ​10^5^ ​CFU/mL) was added to each well. After a 24 ​h incubation, 2 ​μL solution was taken out from each well and was loaded onto an agar plate for growing at 37 ​°C. After 8–24 ​h of growth, the minimum concentration without bacterial growth was determined it as the MIC of PF127-**OA1** against the corresponding bacteria [37].

### 2.4 Time-kill assay

An overnight bacterial culture grown in CAMHB was diluted 1:1000 into the same medium and incubated at 37 ​°C until reaching an OD_600_ of approximately 0.3. This actively growing culture was subsequently diluted in CAMHB to obtain a working suspension containing 5 ​× ​10^5^ ​CFU/mL. The bacterial suspension was then exposed to serially diluted test compounds, with the time of compound addition designated as 0 ​h. At each predetermined time point, 10 ​μL aliquots were withdrawn, serially diluted ten-fold in sterile buffer, and plated on LB agar. After incubation at 37 ​°C overnight, visible colonies were enumerated to determine bacterial viability, expressed as CFU per milliliter.

### 2.5 Effects on bacterial membrane permeability

The overnight culture of *E. coli* or *S. aureus* were harvested, washed, and resuspended in PBS to achieve a final concentration where OD_600_ ​= ​0.1. Subsequently, cells were dispensed into a 96-well plate (200 ​μL per well). **OA1** at indicated concentration were added to the wells, followed by the addition of Propidium Iodide (PI, final concentration ​= ​100 ​μM). The samples were then incubated for 3 ​h at 37 ​°C/220 ​rpm. The fluorescence intensity of PI was observed and recorded using the PE channel on a flow cytometer. The red fluorescence was detected with an excitation of 488 ​nm and an emission of 585 ​± ​30 ​nm. The fluorescence signal was quantified by geometric mean.

### 2.6 Morphology observation using scanning electron microscopy (SEM)

The overnight culture of bacteria was diluted 20 times in CAMHB in culture tubes. **OA1** was added into the culture tubes at indicated concentration. Samples without treatment were served as the negative control. After incubation at 37 ​°C/220 ​rpm for 4 ​h, the samples were centrifugated at 1500 ​g for 12 ​min. The samples were further dehydrated by treating with a series of ethanol/water mixtures (30%, 50%, 70%, 90%, and 100%) and were dried. The samples were mounted on a copper tape, air-dried and sputter-coated with gold for observation using a Hitachi S-4800 field emission scanning electron microscope.

### 2.7 Confocal observation of bacterial membrane permeability

Overnight cultured *E. coli* was suspended in PBS (OD_600_ ​= ​0.5), and PI was added (The final concentration ​= ​100 ​μM) as working solution. Then add the working solution and the specified concentration of **OA1** to the centrifuge tube, using samples without the drug as a control, and incubate them at 37 ​°C for 2 ​h. After incubation, centrifuge the samples at 5000 ​g for 3 ​min, discard the supernatant, and wash the bacteria twice with PBS. Finally, transfer it to a glass slide and imaged using a Nikon A1R MP confocal microscope with a 60× objective lens.

### 2.8 ROS measurement in bacteria

Overnight cultures of *E. coli*, *S. aureus*, *A. baumannii* or *B. subtilis* were harvested, washed, and resuspended in PBS to an OD_600_ of 0.1. The bacterial suspensions were then incubated with 5 ​μM of the fluorescent probe DCFH-DA for ROS detection. Following probe loading, 200 ​μL of each suspension was transferred into individual wells of a sterile 96-well microplate and treated with **OA1** for 3 ​h at 37 ​°C under dark conditions. ROS levels were assessed immediately after treatment using a flow cytometer. Green fluorescence was excited at 488 ​nm and detected at 533 ​± ​30 ​nm. Fluorescence intensity was quantified as the geometric mean of the signal.

### 2.9 Propidium iodide or Ethidium bromide competition assay

A Propidium Iodide or Ethidium bromide (EB) competition assay was used to quantify the DNA-binding affinity of **OA1** The experiment was performed in PBS (pH 7.4) at 25 ​°C. Sheared DNA (10 ​μg/mL) and 4 μg/mL Propidium Iodide or Ethidium bromide were added to a 200 ​μL centrifuge tube. Then, different concentrations of **OA1** were introduced into the centrifuge tubes. The final volume was 200 ​μL. The solution was mixed and incubated at 37 ​°C for 30 ​min. The process was monitored by a microplate reader. Experiments were carried out in duplicate. The *Kd* value determined for Ethidium bromide is 1 ​× ​10^−7^ ​M, for Propidium Iodide is 2.1 ​× ​10^−5^ ​μM [38].

### 2.10 MTT assay

Cytotoxicity of the PF127-**OA1** was determined using the 3-(4,5-dimethylthiazol-2-yl)-2,5-diphenyltetrazolium bromide (MTT) assay with RAW 264.7. Cells were seeded in 96-well plates at 1 ​× ​10^4^ ​cells per well and cultured for 12 ​h in a cell incubator. Subsequently, cells were treated with different concentrations of PF127-**OA1** in DMEM with 10% FBS and then incubated for 24 ​h. Cells with no added compounds were used as controls. After 24 ​h, DMEM medium was removed, cells were washed once with PBS, and then 120 ​μL of fresh medium containing MTT 0.5 ​mg/mL was added. Cells were further incubated in a 37 ​°C incubator for 1.5 ​h. Following this, the DMEM medium was replaced with 100 ​μL of DMSO, and cell viability was determined by measuring the absorbance at 600 ​nm using a microplate reader. Cell viability values were expressed as percentages and calculated as follows: Viability = (OD_600_ nm of treated sample)/(OD_600_ nm of control) ​× ​100%.

### 2.11 Statistical analyses

All experiments were performed with at least three independent replicates (N ​≥ ​3), unless otherwise specified in figure captions or methods. Data are presented as mean ​± ​SD. Statistical significance was determined by Student's t-test with thresholds denoted as follows: *P* ​≤ ​0.05 (∗), ∗*P* ​≤ ​0.01 (∗∗), and *P* ​≤ ​0.001 (∗∗∗).

## 3 Results and discussion

### **3.1** **OA1** has excellent antibacterial performance via multiple mechanisms

We have previously reported a degradable polycationic oligoamidine, **OA1**, as an antibiotic adjuvant working through multimodal antimicrobial mechanisms [29]. However, their antibacterial properties as a standalone agent remained largely unexplored, despite preliminary evidence of antimicrobial activity. To further evaluated the antimicrobial spectrum of **OA1**, we evaluated its antibacterial activity against the most clinically relevant pathogens, ESKAPE. The oligomer showed MICs of 1–8 ​μg/mL (Fig. 1C). Several resistant strains, including methicillin-resistant *S. aureus* (MRSA), *S. aureus* USA300, clinical isolates of *E. faecium*, *S. aureus*, *K. pneumoniae*, *A. baumannii*, *E. coli* and *E. cloacae* were also tested, revealing comparable MICs of 2–8 ​μg/mL. Importantly, **OA1** could maintain its activity in biologically relevant environment. For example, **OA1** maintained strong antimicrobial activity in 50% FBS, with no significant activity loss, demonstrating its stability in protein-rich environments (Fig. 1C).

**Fig. 2:**
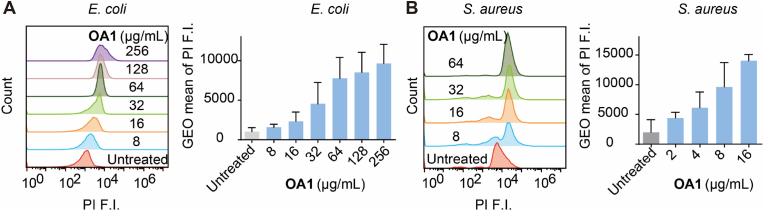
Flow cytometry results of PI assay, indicating the membrane permeability of *E. coli* and *S. aureus* treated by **OA1**.

**Fig. 3:**
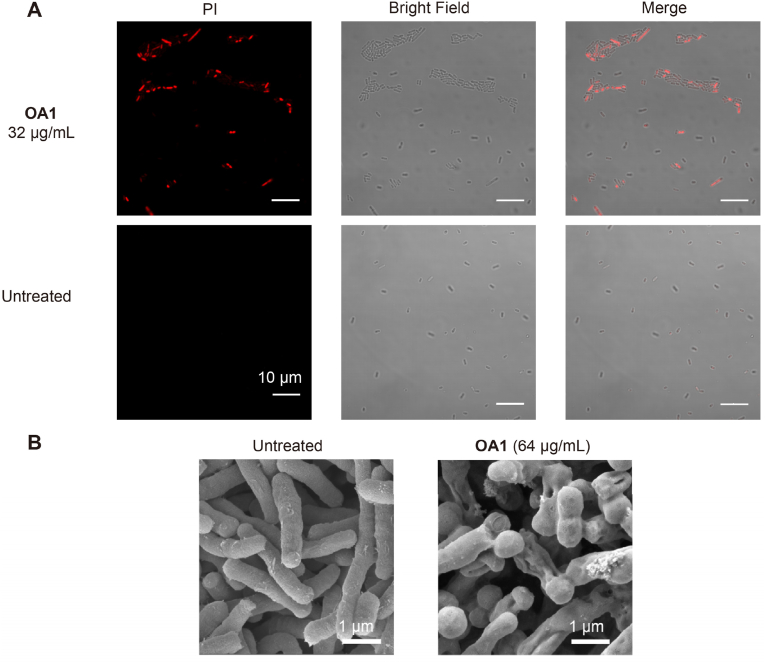
A: Confocal image of *E. coli* stained by PI after **OA1** treatment; B: SEM images of **OA1**-treated *E. coli*, with untreated sample serving as controls.

To verify whether **OA1** exerts antimicrobial effects through membrane disruption, a comprehensive and systematic study is conducted on its interaction with membrane. Firstly, the effect of **OA1** on bacterial membrane permeability was determined by flow cytometry using PI dye. PI is a dye commonly used to study the permeability of cell membrane, as it can enter cells and bind to DNA only when the cell membrane is damaged. We investigated the membrane permeability changes of *E. coli* and *S. aureus* at different **OA1** concentrations. As shown in Fig. 2, **OA1** increased the membrane permeability of *E. coli* at ≥ 8 ​μg/mL and of *S. aureus* at ≥ 2 ​μg/mL, both with clear dose dependence. Meanwhile, confocal microscopy was utilized to observe that upon treatment of *E. coli* with 32 ​μg/mL **OA1** (Fig. 3A), PI entered the bacteria and bound to DNA, emitting red fluorescence. This observation indicated an alteration in bacterial membrane permeability, further supporting the flow cytometry experiment as demonstrated in Fig. 2. Concurrently, SEM was employed to visualize the membrane-damaging effects on bacteria following **OA1** treatment. As shown in Fig. 3B, the surface structure of bacteria changed significantly after **OA1** treatment, resulting in the formation of pores, wrinkles, and folds. Some bacteria even showed severe fractures on their surfaces. These results clearly supported that **OA1** may possess membrane targeting and damaging capabilities.

**Fig. 4:**
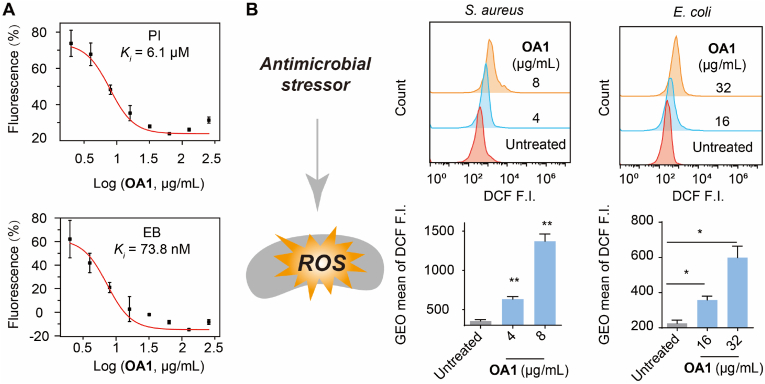
A: Titration curves of **OA1** in PI or EB titration assays, B: ROS generation by **OA1** in *S. aureus* and *E. coli* as probed by DCFH-DA.

In addition to disrupting bacterial membranes, **OA1** is hypothesized to exert antimicrobial activity through additional mechanisms, such as DNA binding. This possibility was investigated using standard DNA-dye displacement assays. PI and EB are two well-established DNA-binding fluorescent dyes, and **OA1** induced a dose-dependent displacement of PI or EB from their interaction with DNA (Fig. 4A). This phenomenon indicates that **OA1** is a strong DNA binder with a higher binding affinity to DNA compared to PI or EB. The inhibition constant (*K*_*i*_) of **OA1** was determined to be 6.1 ​μM for PI-10.13039/100026054DNA and 73.8 ​nM for EB-10.13039/100026054DNA, further supporting its potent DNA-binding capability.

We also observed that **OA1** may impact bacterial redox balance, as **OA1** markedly induced oxidative stress in bacterial cells. We quantified the ROS production levels in different bacteria after being treated with **OA1** (Fig. 4B and S1). The results indicated that all four bacteria producing different degrees of ROS. These findings imply that the antimicrobial effect of **OA1** may be not simply due to membrane damaging, but also caused by DNA binding and the disruption of redox balance. This multi-targeting antimicrobial mechanism of **OA1** may significantly enhance its effectiveness against antibiotic-resistant bacteria.

### **3.2** **OA1** showed effectiveness in different *ex vivo* and *in vivo* infection models

**Fig. 5:**
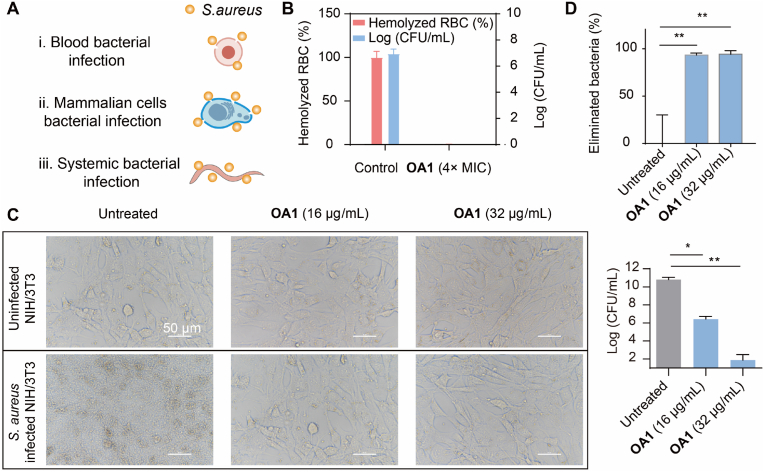
A: Antibacterial effects of **OA1** in both *ex vivo* and *in vivo* infection models; B: Rescue of *S. aureus*-infected RBCs by **OA1**; C: Rescue of *S. aureus*-infected eukaryotic cells (NIH/3T3) with **OA1**; D: Rescue of *S. aureus*-infected *C. elegans* with **OA1**.

When a wound becomes infected, it is not only the wound area that experiences bacterial infection, but there can also be blood infection and systemic infection (Fig. 5A). Based on this, we tested the antibacterial effects of **OA1** in several different infection models. To assess the efficacy of **OA1** in eliminating bacteria from blood and protecting red blood cells (RBCs) from pathogen-induced lysis, and also to evaluate potential hemolytic toxicity concerns associated with the oligoamidine, an *ex vivo* blood infection model was established by incubating *S. aureus* with 4% sheep blood in PBS for 2 ​h at 37 ​°C. Subsequently, **OA1** at a concentration of 4 ​× ​MIC (8 ​μg/mL) was applied for the treatment. Then, the bactericidal efficacy and the hemolysis-rescuing ability of **OA1** were evaluated with this *ex vivo* blood infection model. As shown in Fig. 5B, an infection with *S. aureus* at an initial concentration of 5 ​× ​10^6^ ​CFU/mL led to rapid bacterial proliferation. This uncontrolled bacterial growth was accompanied by complete RBC lysis in the control group. In contrast, **OA1** treatment not only eradicated all *S. aureus*, but also fully preserved RBC integrity without inducing any hemolytic toxicity, demonstrating its potent antimicrobial efficacy and biocompatibility.

When **OA1** was used to treat *S. aureus*-infected NIH/3T3 cells, at concentrations of 16 and 32 ​μg/mL, the load of *S. aureus* was significantly reduced (Fig. 5C and S2). In particular, at the treatment concentration of 32 ​μg/mL, the bacterial load was significantly decreased by 10 LogCFU. Such markedly reduction indicates that **OA1** has strong antibacterial ability. Importantly, it was found that the morphology of cells in the **OA1**-treated group was similar to that of normal cells, whereas the cells of the untreated group exhibited severe damage due to bacterial infection and proliferation. For healthy NIH/3T3 cells, the treatment of **OA1** does not affect their morphology or viability.

To further evaluate the *in vivo* antibacterial efficacy of **OA1**, a *Caenorhabditis elegans* (*C. elegans*) infection model was employed. As shown in Fig. 5D, the infection of *S. aureus* in worms resulted in severe symptoms such as lethargy, cuticle darkening, and complete body rigidity, indicating a substantial bacterial burden and damage. In contrast, worms treated with **OA1** exhibited obvious protecting effects against *S. aureus* infection: **OA1** (32 ​μg/mL) treatment led to an antibacterial effect of up to 98% reduction in bacteria burden, effectively protecting worms from developing characteristic infection symptoms such as body stiffness and locomotor dysfunction. The survival rate of worms was also improved. These findings suggest that **OA1** also shows strong antibacterial activity *in vivo*, mitigating bacterial-induced damage to the host. Taken together, our *ex vivo* and *in vivo* results further support that **OA1** shows a great application potential as an active antibacterial agent for modulating a biodegradable and biocompatible antimicrobial hydrogel.

### 3.3 Pluronic F-127 could integrate **OA1** to form antimicrobial hydrogel with excellent performance

**Fig. 6:**
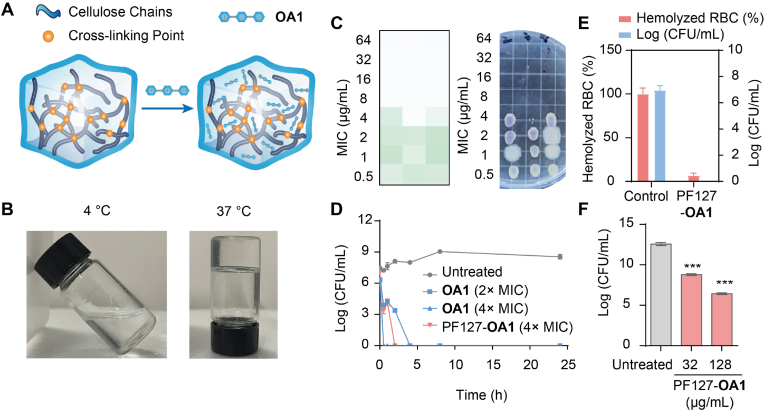
A: PF127-**OA1** Preparation Diagram; B: The morphology of PF127-**OA1** at different temperatures; C: MIC of PF127-**OA1** against *S. aureus* (The darker color represents a weaker antimicrobial effect); D: Bactericidal kinetics of PF127-**OA1** against *S. aureus*; E: Rescue of *S. aureus*-infected RBCs by PF127-**OA1** (4 ​× ​MIC); F: Rescue of *S. aureus*-infected eukaryotic cells (RAW 264.7) with PF127-**OA1**.

As we had demonstrated the excellent antibacterial activity of **OA1**, we then sought to integrate this oligomer into a hydrogel matrix to generate an antimicrobial hydrogel for effective topical application on skin wounds. The normal human skin temperature on the trunk ranges from 33.5 to 36.9 ​°C. Based on this, we chose a thermosensitive Pluronic F-127 (PF127) hydrogel as the matrix for loading **OA1** (Fig. 6A). PF127 dissolves in water as a single molecular chain at low temperatures (below 15 ​°C). When the temperature rises to the critical micelle temperature (CMT ​≈ ​14 ​°C), the hydrophobic PPO segments dehydrate and aggregate to form spherical micelles. As the temperature continues to increase to the low gelation temperature (LGT ​≈ ​20 ​°C), the micelles closely pack into a cubic lattice structure, forming a physically cross-linked network that macroscopically appears as a gel. At physiological temperatures (>LGT), PF127 rapidly gels, exhibiting plastic deformation, which allows the material to conform to skin deformations without breaking [39]. First, we characterized the gelation behavior of the formulating PF127-**OA1** hydrogel. As shown in Fig. 6B, the PF127-**OA1** hydrogel undergoes sol-to-gel transition at 35 ​°C, confirming its suitability for topical applications as it forms gelling upon administration and thus the contact with tissues at the wound areas can be maximized. We then assessed the impact of PF127-**OA1** hydrogel on cellular viability in RAW 264.7 ​cell cultures. As shown in Fig. S3, cell viability assays performed after 24 ​h incubation demonstrated that the PF127-**OA1** combination was safe for the cells, leading to well-preserved cell viability (>90% survival rate) at MIC concentrations.

The antibacterial performance of the PF127-**OA1** hydrogel was also studied (Fig. 6C). Bactericidal kinetics against *S. aureus* demonstrated that the PF127-**OA1** hydrogel showed fast-killing antimicrobial kinetics, although slightly slower than free **OA1** likely due to the amphiphilic masking effect of PF127 matrix (Fig. 6D). Furthermore, in RBC-bacterium coculture model, treatment with PF127-**OA1** hydrogel demonstrated a significant reduction of bacterial load and effective protection of RBCs from hemolytic damage (Fig. 6E). The mammalian cell-bacterial co-culture system also confirmed that the hydrogel showed robust antibacterial activity and cellular protective effects against bacterial infection (Fig. 6F and S4). The PF127 system loaded with **OA1** significantly delayed the drug release rate [40]. Although this sustained release effect somewhat reduced the immediate antibacterial efficacy of PF127-**OA1** against eukaryotic cells infected with *S. aureus* (Fig. 5, Fig. 6F), it significantly enhanced the sustained therapeutic effect on infected cells by prolonging the action time of **OA1**. These collective findings may verify that PF127-**OA1** hydrogel has potent antimicrobial activity and excellent biocompatibility for therapeutic applications.

### 3.4 The antibacterial effect of **OA1** in *ex vivo* model

**Fig. 7:**
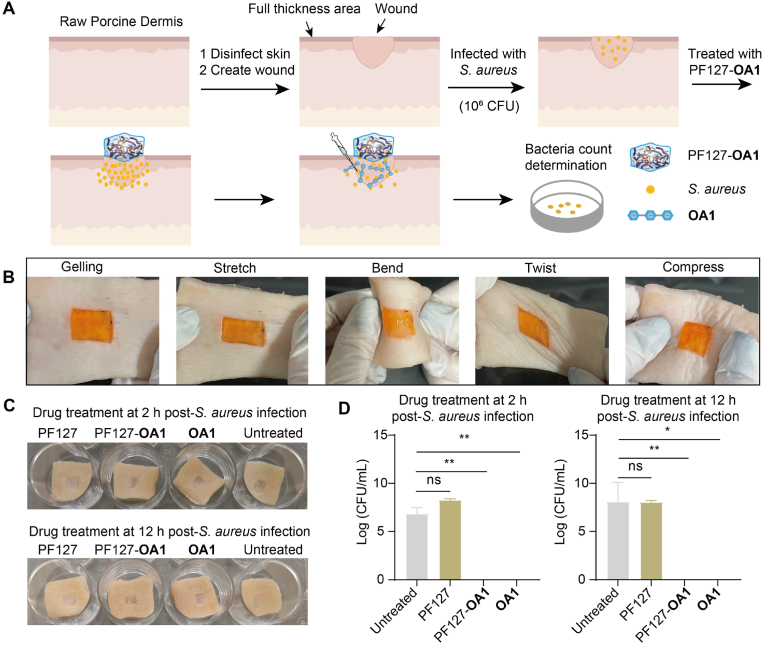
A: Schematic diagram of pig skin *ex vivo* infection model; B: Photographs of the stretching, bending, twisting and compressing processes of thermogel; C and D: **OA1** (256 ​μg/mL) and PF127-**OA1** (256 ​μg/mL) hydrogel rescue pig skin *ex vivo* infection model.

Encouraged by the excellent *ex vivo* effects of PF127-**OA1** hydrogel, the antibacterial activity of this hydrogel on wound infections was systematically evaluated using an *ex vivo* pig skin model (Fig. 7A). At physiological temperature (37 ​°C), the pre-cooled PF127-**OA1** solution underwent a phase transition, forming a stable gel at the wound site. Notably, even when this antimicrobial gel was subjected to mechanical stress such as stretching, bending, torsion, and compression, the hydrogel remained firmly adhered to the wound surface, ensuring sustained local drug delivery (Fig. 7B).

To further assess its antibacterial efficacy, the PF127-**OA1** hydrogel was applied to infected pig skin, and the bacterial growth was monitored over a 24 ​h treatment period. These results showed that the hydrogel exhibited a remarkable ability to suppress *S. aureus* proliferation compared to both the untreated group and the blank hydrogel control group (Fig. 7C, D and S5). Notably, after 24 ​h of treatment, the PF127-**OA1** hydrogel completely eradicated *S. aureus*, achieving a statistically significant antibacterial effect. In contrast, PBS buffer alone did not form a gel on the skin, providing minimal wound protection, whereas the blank PF127 hydrogel formed a gel-like layer that maintained a moist wound environment, which actually helped *S. aureus* as the *S. aureus* CFU was determined to be higher than that of the PBS buffer group. These findings highlight the potent antibacterial potential of the PF127-**OA1** hydrogel and demonstrate its strong ability to eliminate *S. aureus* while maintaining a stable and adherent gel structure on the wound surface.

## 4 Limitation

In this study, PF127-**OA1** hydrogel exhibits excellent antibacterial *in vitro*. However, its internal performance, particularly the stability of wound adhesion and the lasting antibacterial effect, needs to be verified. Although the experimental conditions were precisely controlled (such as maintaining a physiological temperature of 37 ​°C) to replicate the *in vivo* microenvironment, the complexity of real biological systems far exceeds that of *in vitro* models. Therefore, subsequent research urgently needs to systematically evaluate the performance of PF127-**OA1** *in vivo* using small animal skin defect infection models.

## 5 Conclusions

In summary, we have developed a biodegradable and biocompatible oligomer (**OA1**) showing potent antimicrobial activity as a standalone agent, and demonstrated that **OA1** was able to damage bacterial membranes, target bacterial DNA, and induce ROS stress in bacteria, leading to multi-form of damage to the bacterial cells for improved anti-resistance effectiveness. We also demonstrated that **OA1** could be easily formulated with a thermosensitive hydrogel PF127 to produce a ready-to-use thermo-responsive antimicrobial hydrogel with excellent antibacterial performance, which was validated in both *in vitro* and *ex vivo* models. Compared to most reported antimicrobial hydrogels produced by direct crosslinking of antimicrobial polymers, our newly developed PF127-**OA1** hydrogel provides more advanced features including the unique thermosensitive property, allowing easy and effective administration, maximizing wound protection, and controlled releasing of **OA1** for therapeutic applications.

## CRediT authorship contribution statement

**Min Wang:** Writing – original draft, Validation, Formal analysis. **Xinyun Zeng:** Writing – original draft, Data curation. **Xiuping Wang:** Investigation. **Zhiyuan Zhang:** Software. **Siwei Guo:** Funding acquisition. **Yang Deng:** Funding acquisition. **Xin Li:** Funding acquisition. **Lin Yao:** Funding acquisition. **Jiaqi Li:** Software. **Wing-Leung Wong:** Writing – review & editing. **Yugang Bai:** Writing – review & editing. **Xinxin Feng:** Writing – review & editing, Resources, Project administration.

## Ethics approval

All the animal studies were performed in accordance with the national and provincial regulations on animal studies. The certificate for the use of animals for research was SYXK (Hunan) 2022–0007, licensed by the Department of Science and Technology of Hunan Province on April 20, 2022, valid for 5 years. The protocols were approved by the Laboratory Animal Welfare and Ethics Review Committee, Hunan University.

## Declaration of generative AI in scientific writing

Not applicable.

## Funding information

The funding support from the 10.13039/501100012166National Key Research and Development Program of China (2023YFD1800100 to X.F. and Y. B.), 10.13039/501100001809National Natural Science Foundation of China (Grants 22177031 to X.F., 92163127 to Y.B.), 10.13039/501100004735Natural Science Foundation of Hunan Province (2024JJ4007 to X.F., 2024JJ2010 to Y.B), Science Fund for 10.13039/501100019338Distinguished Young Scholars of Hunan Province (2024RC3078 to X.F., 2022RC1107 to Y.B), Hunan Provincial Key Laboratory of Anti-Resistance Microbial Drugs, the Third Hospital of Changsha (2023TP1013 to X.F.), Cross fusion research project of Air Force Medical University (2024JC051 to X.F.), the Health and Medical Research Fund (HMRF), Hong Kong SAR (22210412 to Wong WL), Hunan Provincial Innovation Foundation For Postgraduate (QL20220075 to J. L.) are gratefully acknowledged.

## Data availability

All data supporting the findings of this study are included in this published article and its supplementary information files.

## Declaration of competing interest

The authors declare that they have no known competing financial interests or personal relationships that could have appeared to influence the work reported in this paper.

## Appendix A Supplementary data

Multimedia component 1 Supporting Information is available and includes detailed experiment protocols and additional experiment results. Multimedia component 1
