# Supplementary material for: A biodegradable antimicrobial oligomer-containing hydrogel for drug-resistant bacteria-infected skin wound treatment
Source: Pharm Sci Adv. 2025 Aug 22;3:100091. doi: 10.1016/j.pscia.2025.100091 (PMC12709906; doi:10.1016/j.pscia.2025.100091)
Supplement: Multimedia component 1 — Supporting Information is available and includes detailed experiment protocols and additional experiment results. [file mmc1.docx]

Supplementary Materials for

**A biodegradable antimicrobial oligomer-containing hydrogel for drug-resistant bacteria-infected skin wound treatment**

**This PDF file includes:**

Experimental Section

Supplementary Figures

Experimental Section

*General*

Unless otherwise stated, all chemical reagents were of analytical grade, obtained from Shanghai Adamas Reagent, Ltd. or Shanghai Macklin Biochemical Co., Ltd. and were used without further purification. All other solvents were dried and stored over activated 4 Å molecular sieves.

Cells

Standard bacterial strains such as *E. coli* (ATCC 29425), *S. aureus* Newman strain (ATCC 25904), *A. baumannii* (Bouvet and Grimont, ATCC 19606) were kindly offered by Professor Douglas A. Mitchell in University of Illinois at Urbana-Champaign. Clinical strains (*S. aureus-1* (sklx001913)*, S. aureus* (USA300)*, MRSA* (13-27)*, K. pneumoniae-1* (sklx001877)*, K. pneumoniae-2* (sklx001855)*, E. coli-1*(sklx001912)*, E. coli-2* (sklx001918)*, E. cloacae* (sklx001892)) were provided by Dr. Kai Zhou in The First Affiliated Hospital of Southern University of Science and Technology and Dr. Cuiyan Tan in Department of Pulmonary and Critical Care Medicine Fifth Affiliated Hospital of Sun Yat-sen University. All bacterial strains were confirmed by 16S DNA sequencing. Murine embryonic fibroblast cell line NIH/3T3 (ATCC CRL-1658) and Murine macrophage cell line RAW 264.7 (ATCC TIB71) was purchased from ATCC and incubated with 5% CO_2_ until a monolayer with over 80% confluence was obtained. *C. elegans* N2 strain was a kind gift from Dr. Haijun Tu in Hunan University, China.

*Instrumentation*

**Scanning Electron Microscopy (SEM).** SEM experiments were conducted on a Hitachi S-4800 scanning electron microscope. Bacteria sample were gold-plated before they were observed on SEM.

**Flow Cytometry Study.** All the flow cytometry studies were performed on a Becton Dickinson Accuri C6 Plus instrument. The data was processed in FlowJo, aligned and annotated in Adobe Illustrator CC.

**Confocal Microscopy.** Fluorescence microscope images were acquired using a Nikon Eclipse Ti2-E Laser Confocal Microscope. For blue fluorescence, wavelength was set at 405 nm for excitation. For green fluorescence, wavelength was set at 488 nm for excitation. For red fluorescence, wavelength was set at 561 nm for excitation.

*Ex vivo and in vivo models*

**RBC-bacterium coculture model**

A *S. aureus* was added to 4% sheep blood in PBS to a final concentration of 5 × 10^5^ CFU/mL. Samples were then treated with **OA1**, PF127-**OA1** at indicated concentration, or PBS and incubated

for 24 h at 37℃. Bacteria loading was determined by plating an aliquot of sample (2 μL) with different serial dilutions onto an agar plate, and CFUs were counted after a 12 h incubation at 37℃.

To determine the percent hemolysis, samples were centrifuged, and the absorbance of the supernatant at 576 nm was measured using a Triton X-100-treated blood sample as the positive control (100%

hemolysis) and a nontreated blood sample as the negative control (0% hemolysis).

**Mammalian-bacterium co-culture model**

In a 12-well plate, NIH/3T3 cells (2 × 10^4^ cells per mL) or RAW 264.7 cells were cultured in DMEM supplemented with 10% FBS in a humidified atmosphere with 5% CO_2_ at 37°C. Cells were maintained for 12 h to reach a confluent monolayer. Medium was removed from wells followed by addition of DMEM + 10% FBS containing ~10^8^ CFU of *S. aureus*, with **OA1**, PF127-**OA1** or without drugs. Bacteria were not added to the control sample. The cell or cell-bacteria mixtures were then cultured at 37°C for 24 h before the samples were imaged using an optical microscope (Nikon Ti-S, Nikon Co., Tokyo, Japan). The cultures were then plated onto agar plates. The CFUs were counted after incubation at 37°C overnight.

***C. elegans* model for antimicrobial efficacy**

*C. elegans* N2 were maintained at 20°C following above standard procedures. *S. aureus* was used for *C. elegans* infection. *S. aureus* was grown in CAMHB medium to stationary phase. One hundred fifty microliters of overnight bacterial cultures were inoculated on modified NGM with 0.35% peptone to obtain a lawn of bacteria. Adult worms were then transferred to the NGM plates for infection. After 12 h of infection, worms were collected and washed with PBS buffer for three times, before 10 worms were transferred to a centrifuge tube. Worms were incubated with **OA1** or PBS (negative control). After a 12 h treatment, worms were washed three times with PBS buffer and examined for morphological changes and viability. The worms were then ground with a glass rod and samples were serially diluted and plated onto LB agar plates containing selection antibiotics (20 μg/mL Nalidixic acid for *S. aureus*). Plates were incubated at 37°C for 17 h before CFUs were determined. The bacterial CFUs were then divided by the number of worms in each treatment. The log reduction in bacterial growth upon drug treatment or PBS-treated worms was then calculated.

**Antimicrobial efficacy evaluation on *ex-vivo* wound model**

With slight modiﬁcation, the *ex-vivo* experimental method was performed as previously described^[1]^. The pigskin used in this study was shaved and frozen at −4 ℃. During the experiment, the remaining fat layer was removed from the frozen skin slices with a scalpel, and the skin was further cut into 1.5 × 1.5 cm small pieces. The small pieces were disinfected with 75% ethanol, washed with sterile ultrapure water, wiped dry with sterile (Autoclaved) paper towel, and put into a 12 well plate. The epidermis was removed leaving a dermal wound about 2-3 mm deep and 5 mm in diameter. The skin area (including the wound) was washed with sterile ultrapure water.

The samples used for Firmness test were stained (for better visibility) and injected into the wound, then incubated at 37℃ to form a rigid gel. Then, the adhesion of PF127-**OA1** hydrogel was examined under tension (bending at the ends of the skin), bending, torsion, and compression.

For the bacterial clearance of *S. aureus* from infected pig skin wounds experiment, *S. aureus* cultured overnight was diluted 1000 times with fresh LB medium and 10 µL of the bacterial culture was added to each wound area to allow bacteria to adhere to the skin for 2 h or 24 h. Then, 50 µL PBS, PF127, or PF127-**OA1** were applied to infected wounds. After incubating the plate at 37℃ for 24 h, the bacteria were harvested by gently rubbing the wound and adding 50 µL PBS. Bacterial plate count method was used to evaluate the remaining bacteria in each group.


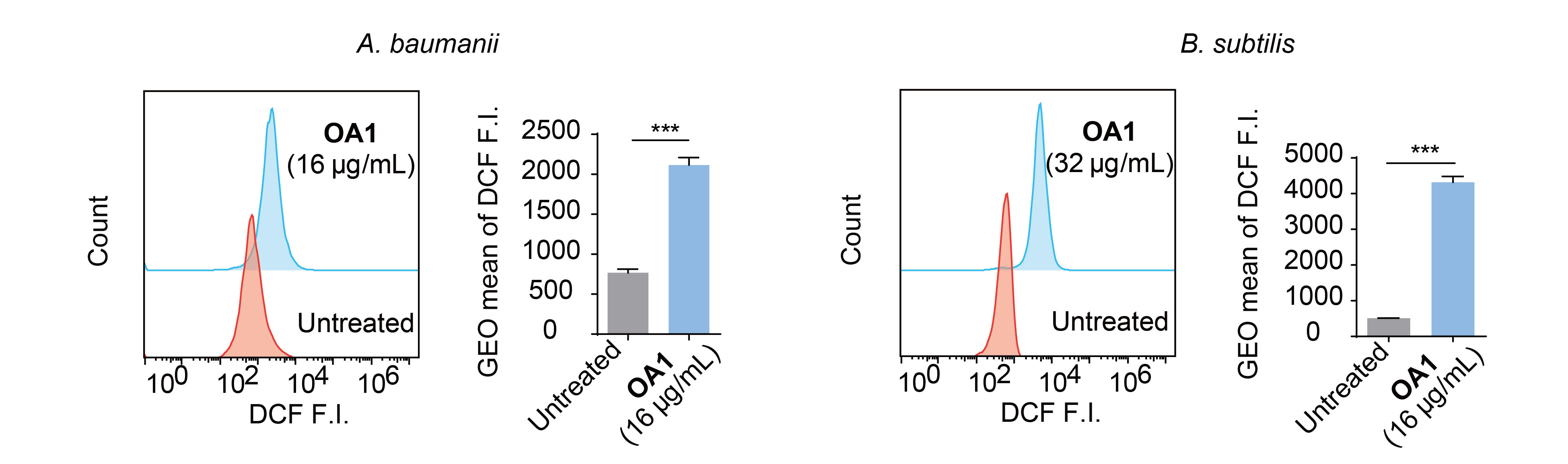


Fig. S1. ROS generation by OA1 in *A. baumannii* and *B. subtilis* as probed by DCFH-DA.


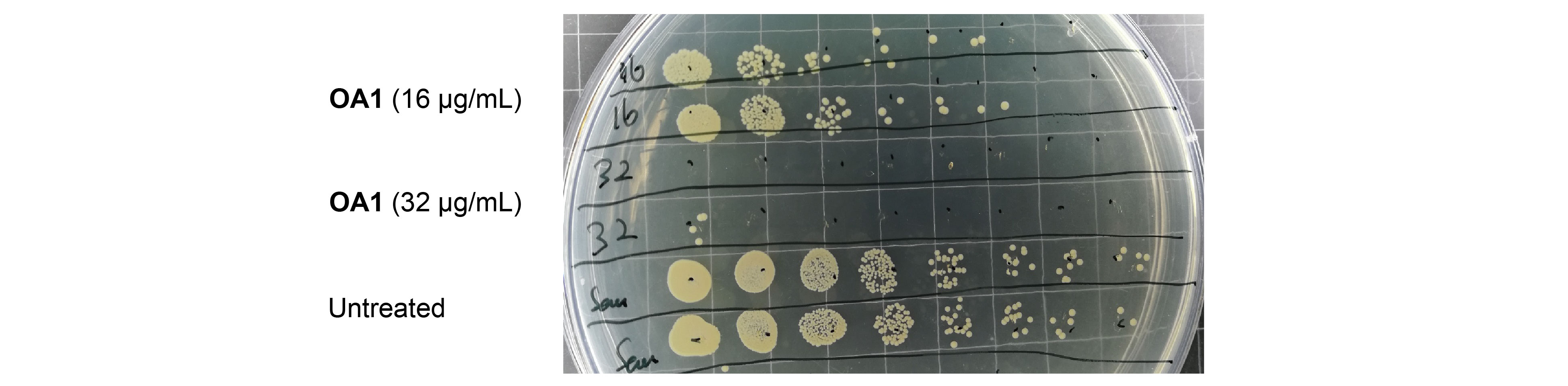


Fig. S2. Rescue of *S. aureus*-infected eukaryotic cells (NIH/3T3) with **OA1**.


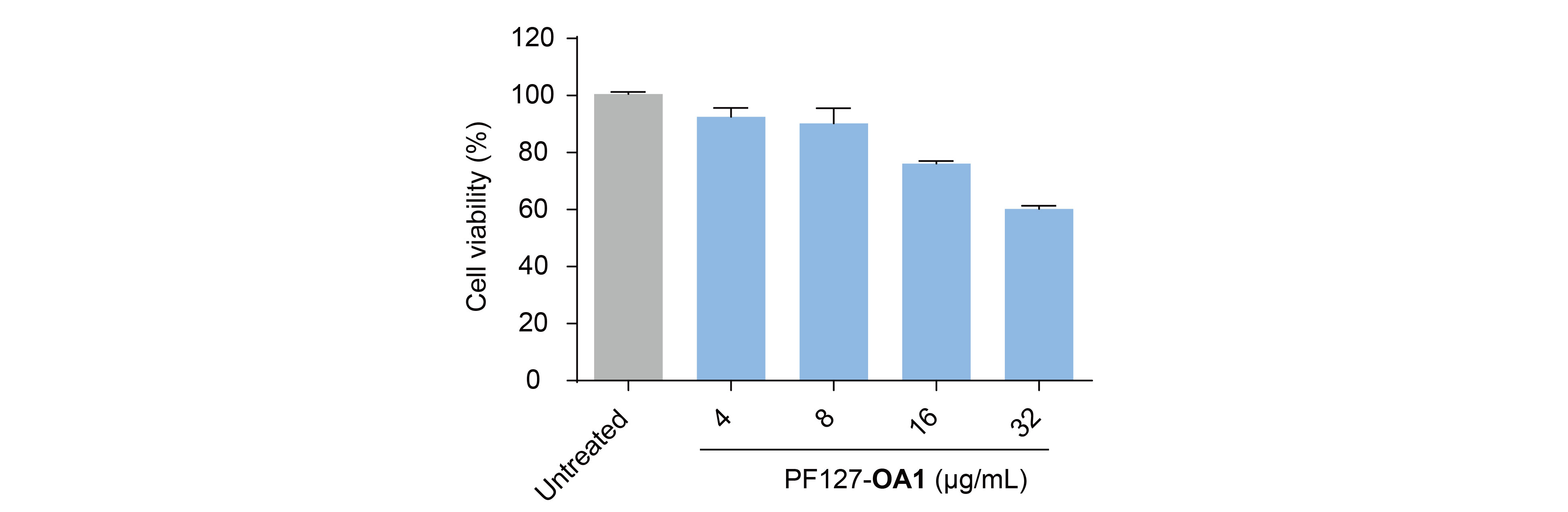


Fig. S3. Cytotoxicity studies of PF127-**OA1** at various concentrations against RAW 264.7 cells.


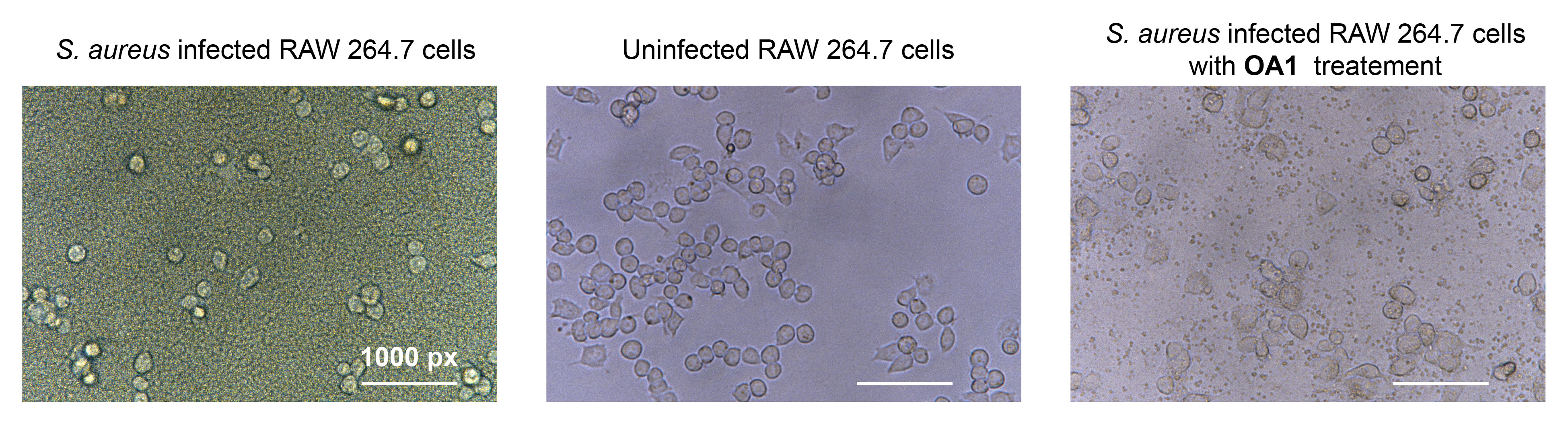


Fig. S4. Rescue of *S. aureus*-infected eukaryotic cells (RAW 264.7) with PF127-**OA1**.


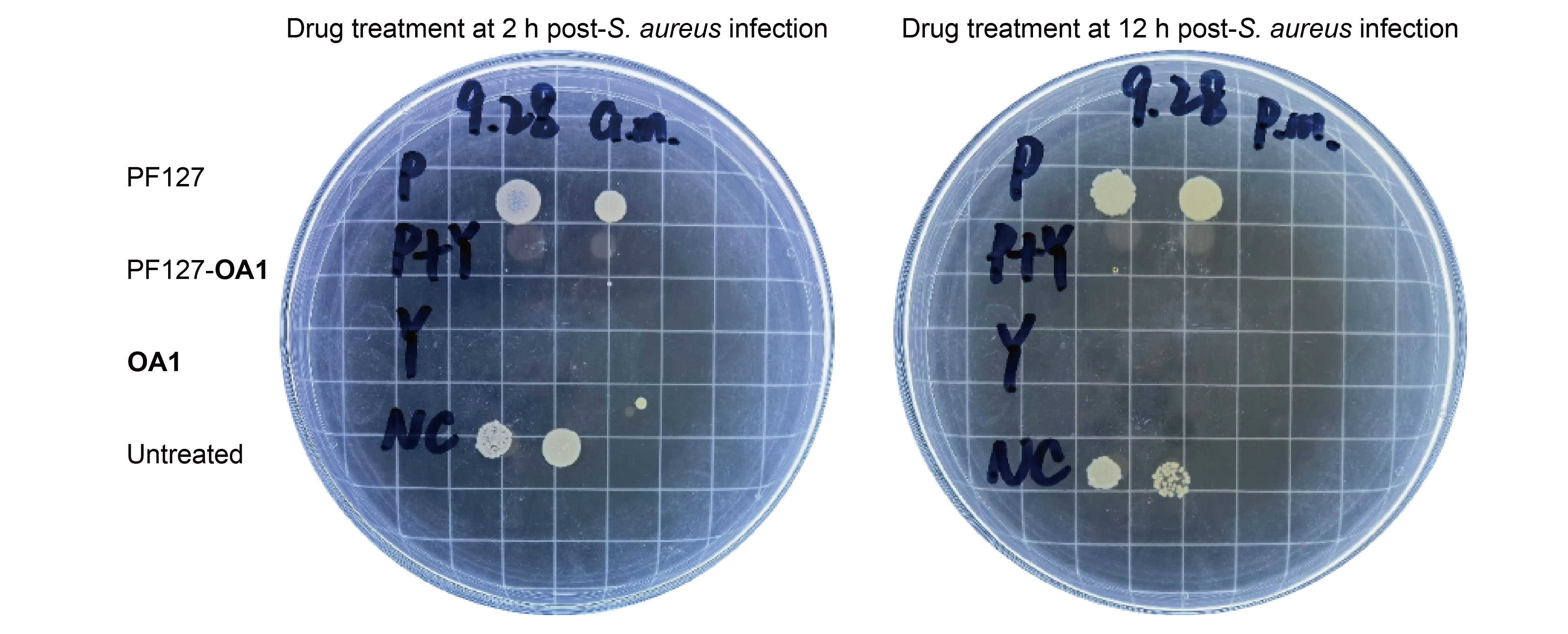


Fig. S5. **OA1** (256 μg/mL) and PF127-**OA1** (256 μg/mL) hydrogel rescue pig skin *ex vivo* infection model.

**Reference**

[1] C. Li, R. Nyaruaba, X. Zhao, H. Xue, Y. Li, H. Yang, H. Wei, Thermosensitive Hydrogel Wound Dressing Loaded with Bacteriophage Lysin LysP53, Viruses 14 (2022) 1956. https://doi.org/10.3390/v14091956.
